# Development and Validation of Nomograms Predicting the Overall and the Cancer-Specific Survival in Endometrial Cancer Patients

**DOI:** 10.3389/fmed.2020.614629

**Published:** 2020-12-23

**Authors:** Xingchen Li, Yuan Fan, Yangyang Dong, Yuan Cheng, Jingyi Zhou, Zhiqi Wang, Xiaoping Li, Jianliu Wang

**Affiliations:** ^1^Department of Obstetrics and Gynecology, Peking University People's Hospital, Beijing, China; ^2^Beijing Key Laboratory of Female Pelvic Floor Disorders Diseases, Beijing, China

**Keywords:** endometrial cancer, prognostic factor, cancer-specific survival, nomogram, SEER database

## Abstract

**Background:** The present study was aimed at developing nomograms estimating the overall survival (OS) and cancer-specific survival (CSS) of endometrial cancer (EC)-affected patients.

**Patients and Methods:** We retrospectively collected 145,445 EC patients between 2004 and 2015 from the Surveillance, Epidemiology, and End Results (SEER) database. Independent prognostic factors were identified via univariate and multivariate Cox analyses. These risk factors were used to establish nomograms to predict 3- and 5-year OS and CSS rates. Internal and external data were used for validation. The predictive accuracy and discriminative ability were measured by using concordance index (C-index) and risk group stratification.

**Results:** A total of 63,510 patients were collected and randomly assigned into the training cohort (*n* = 42,340) and the validation cohort (*n* = 21,170). Age at diagnosis, marital status, tumor size, histologic type, lymph node metastasis, tumor grade, and clinical stage were identified as independent prognostic factors for OS and CSS (*p* < 0.05 according to multivariate Cox analysis) and were further used to construct the nomograms. The area under the receiver operating characteristics (ROC) curve was greater than that of International Federation of Gynecology and Obstetrics (FIGO) staging system for predicting OS (0.83 vs. 0.73, *p* < 0.01) and CSS (0.87 vs. 0.79, *p* < 0.01) in the training cohort. The stratification into different risk groups ensured a significant distinction between survival curves within different FIGO staging categories.

**Conclusion:** We constructed and validated nomograms that accurately predicting OS and CSS in EC patients. The nomograms can be used for estimating OS and CSS of individual patients and establishing their risk stratification.

## Introduction

Endometrial cancer (EC) is the most common gynecologic malignancy in the United States (1), with an increased number of cases also in China, especially in young women (2). Several prognostic risk factors have been associated with the overall survival.

The most commonly used classification system in EC patients is the International Federation of Gynecology and Obstetrics (FIGO) staging system, in which patients with non-metastatic cancer are stratified based on the depth of myometrial invasion, cervical stromal invasion, as well as the extent of lymph node involvement (3). However, survival of patients with the same clinical stage varies widely because this system considers the survival rate independently from other prognostic factors such as age, tumor grade, and histologic type (4, 5).

Nomogram is a statistical prediction tool that incorporates all prognostic factors to calculate the risk score of each patient and estimate the survival outcome for the individual, as has been widely demonstrated in other cancers such as breast cancer, gastric cancer, and ovarian cancer (6–8). Therefore, considering the advancements in individualized cancer treatments, a more precise survival prediction model for EC patients need further investigation. There are several established nomograms predicting lymph node metastasis (9), recurrence (10), and lymphatic dissemination (11) in EC. Nomograms for predicting long-term survival outcome in EC are limited, and the most extensive used and validated nomogram is the Memorial Sloan-Kettering Cancer Center (MSKCC) (12). However, novel characteristics have been described from then. To our knowledge, a nomogram which includes available prognostic factors and that is based on a large cohort to predict the overall survival (OS) and cancer-specific survival (CSS) of EC patients has not recently been reported yet.

The Surveillance, Epidemiology, and End Results (SEER) database collects data from 18 cancer registries and covers about 30% of the US population (13). Using the SEER database, we established the OS and CSS nomograms on a nationwide, population-based cohort in EC patients from 2004 to 2015 in the current study. The nomograms incorporated independent prognostic factors for survival identified in EC, and precisely predicted 3- and 5-years overall and cancer-specific survival of individual EC patients. These nomograms contributed to a better risk stratification and clinical decision making.

## Materials and Methods

### Patients Cohort and Variables

Patients information was acquired by the National Cancer Institute SEER database, which was accessed via the SEER^*^Stat software (Version 8.3.5; NCI, Bethesda, MD). We identified patients who underwent primary surgery for FIGO 2009 staging system that were diagnosed between 2004 and 2015. The inclusion criteria included the following: age at diagnosis > 18 years; endometrial cancer pathologically confirmed by histology (histological code: 8140-8389 for EEA, 8440-8499 for SEA). For patients who had a history of prior malignancy and/or radiotherapy before surgery, were excluded. Clinical pathological features including patient age, year of diagnosis, marital status, tumor size, histological type, myometrial invasion, cervical stromal invasion, lymph node metastasis, tumor grade, clinical stage, chemotherapy, radiotherapy, time of survival, OS, and CSS were collected in the study. In addition, cases were excluded from the study if they had incomplete information on any of these characteristics. Age of the patients was stratified into five groups from 41 to 70 with a 10-year interval. Marital status was classified as married, single, and others (including widowed and divorced). The histological types were classified as endometrial endometrioid adenocarcinoma (EEA, ICD-O-3 codes: 8140-8389), serous endometrioid adenocarcinoma (SEA, ICD-O-3 codes: 8440-8499), and other types. Tumor size was categorized as ≤ 2, 2.1–5, 5.1–10, and >10 cm. Race was excluded because ethnicity proportions in the SEER database was uneven, which would bring bias to the nomogram. The eligible patients were randomly divided into the training cohort and the validation cohort according to the ratio of 2–1.

### Construction and Validation of the Nomogram

Univariate and multivariate Cox regression analyses were used to identify the prognostic factors of the OS and the CSS in the training cohort. The independent risk factors were used to build the nomogram for the OS and CSS, respectively. The nomogram performance was assessed in both the training and validation groups by calculating discrimination and calibration criteria (14). An index of concordance (C-index) between predicted probability and observed outcome was calculated to evaluate the predictive performance. C-index value ranged from 0.5 to 1.0, with 0.5 indicating a random likelihood and 1.0 indicating a perfect match. Generally, a C-index value of 0.7 or higher suggests an acceptable fit (15). Patient differentiation was determined using the area under the receiver operating characteristic (ROC) curve. The area under the ROC curve (AUC) is a summary measure of the ROC that reflects the ability of a test to discriminate the outcomes across all possible levels of positivity. The AUC ranges from 0 to 1, and a model is considered to have a poor, fair, or good performance if the AUC lies between 0.5 and 0.6, 0.6 and 0.7, or is >0.7, respectively. The AUC of clinical stage was compared with that of the nomogram in both the OS and the CSS group. A calibration plot was generated to visualize how far the predictions were from the actual outcomes, displaying mean nomogram-based predictions in the training and validation cohorts on the horizontal axis vs. the actual observed survival probabilities.

### Risk Group Stratification Based on the Nomogram

In addition to numerically comparing the discrimination ability of the nomogram, we evenly grouped the patients into five risk groups according to the risk score calculated by the nomograms (from the highest to the lowest) in the training set. These values were then applied to the validation set and the respective Kaplan-Meier survival curves were determined.

### Statistical Analysis

All the categorical variables were described as frequencies and percentages. Baseline of the clinical and the pathological characteristics of the patients were compared between the training and the validation cohort using a Chi-square or Fisher exact tests as appropriate. Univariate and multivariate Cox regression analyses were performed using the SPSS 22.0 (IBM Corp, USA). All variables that in univariate analysis showed a *p-*value < 0.05 were selected for the multivariate analysis. Hazard ratios (HR) and corresponding 95% confidence interval (CI) of variables were also calculated. The independent factors were used to build the nomograms for 3- and 5-year OS and 3- and 5-year CSS. Calibration curves were constructed to compare consistency between predicted and observed survival. Construction of the nomogram, calibration, and survival curves were performed using the *rms, foreign*, and *survival* packages in R, version 3.5.1 (http://www.r-project.org).

## Results

### Screening Process and Demographic Characteristics of Patients

**Figure 1:**
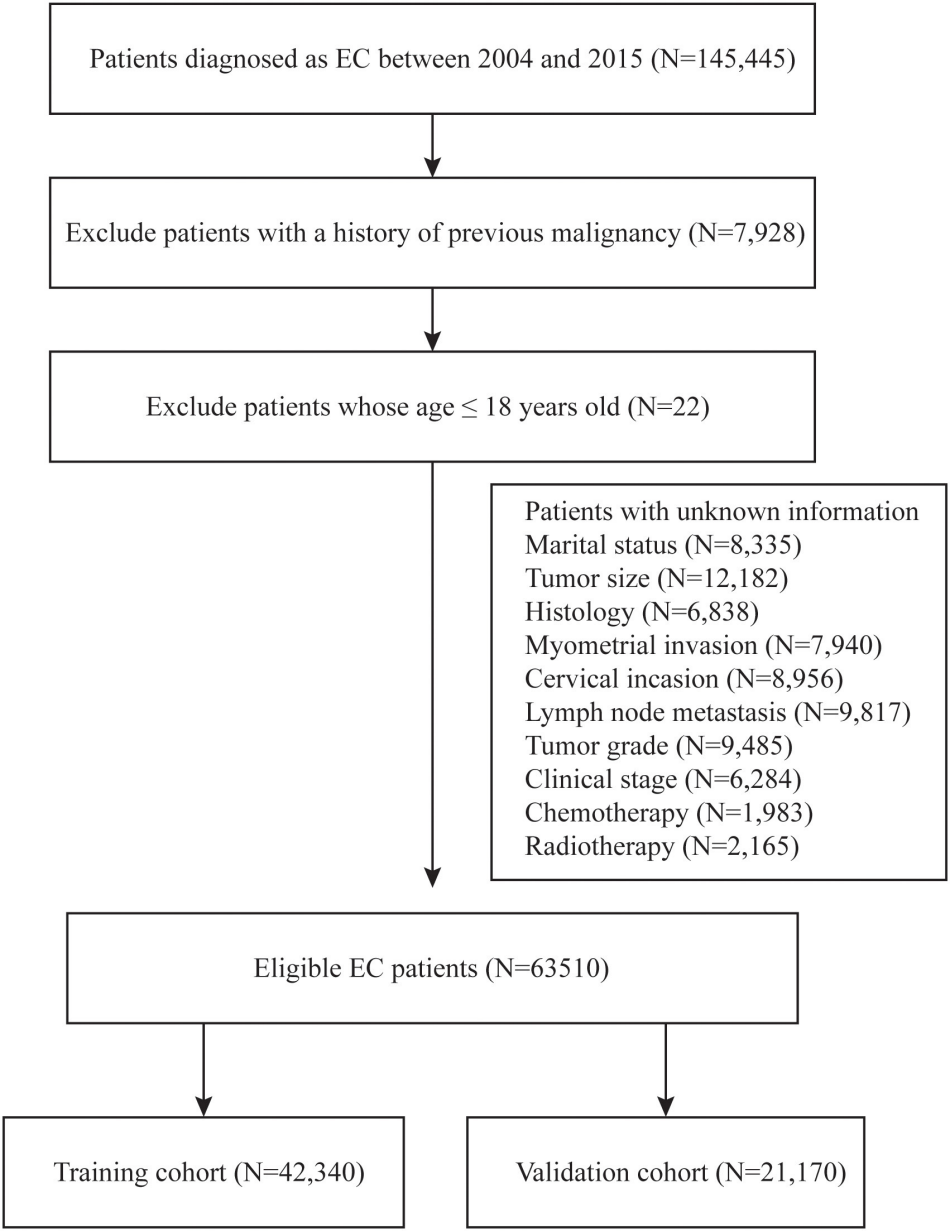
Flow diagram of inclusion criteria and exclusion criteria. According to the criteria, 145,440 patients were collected from the SEER database and randomly assigned into the training cohort (*N* = 42,340) and validation cohort (*N* = 21,170).

During the 2004–2015 time period, a total of 145,445 patients with endometrial cancer were identified from the SEER database. Of these, 63,510 patients were enrolled in this study according to the inclusion criteria and exclusion criteria listed in Figure 1. These eligible patients were randomly divided into the training cohort (*n* = 42,340) and the validation cohort (*n* = 21,170). The demographic characteristics of the patients were listed in Table 1. In the training cohort, the mean age of the patients was 62.43 ± 11.66 years old. The mean tumor size was 6.56 ± 15.60 cm. For the marital status, a total of 22,726 (53.68%) patients were married, while 8,168 (19.29%) patients were single. Among these patients, 37,041 (87.48%) patients were diagnosed with EEA, 3,185 (7.52%) patients with SEA, and 2,114 (4.99%) patients were diagnosed with other cancer types. The myometrial invasion of most patients (27,721, 65.74%) was <50%. Most people were negative for cervical stromal invasion (33,797, 79.82%) and lymph node metastasis (37,500, 88.57%). As far as the clinical stage aspect, 70.46% people were at stage I, 8.17% were at stage II, 15.64% were at stage III, and 5.73 were at stage IV. The median survival time was 61.17 ± 40.99 months. The rest of the clinicopathologic characteristics of the patients in the training and validation group are listed in Table 1. There was no significant difference regarding all the enrolled variables between the two cohorts.

**Table 1:** Demographic characteristics of patients in training and validation cohorts.

	**Training cohort**	**Validation cohort**	***p*-value**
Total number	42,340	21,170	
	**Mean** **+** **SD**	**Mean** **+** **SD**	
Age (years)	62.43 ± 11.66	62.37 ± 11.54	0.859
Tumor size (cm)	6.56 ± 15.60	6.62 ± 15.76	0.472
Survival months	61.17 ± 40.99	61.17 ± 40.98	0.916
	***N*** **(%)**	***N*** **(%)**	
Age (years)			0.711
≤ 40	1,497 (3.54)	719 (3.40)	
41–50	4,241 (10.02)	2,164 (10.22)	
51–60	13,001 (30.71)	6,553 (30.95)	
61–70	13,533 (31.96)	6,719 (31.74)	
>70	10,068 (23.78)	5,015 (23.69)	
Year of diagnosis			0.921
2004–2007	10,840 (25.60)	5,410 (24.28)	
2008–2011	14,395 (34.00)	7,193 (33.98)	
2012–2015	17,105 (40.40)	8,567 (41.47)	
Marital status			0.105
Married	22,726 (53.68)	11,454 (54.10)	
Single	8,168 (19.29)	4,046 (19.11)	
Others	11,446 (27.03)	5,670 (26.78)	
Tumor size (cm)			0.243
≤ 2	10,437 (24.65)	5,171 (24.43)	
2.1–5	20,917 (49.40)	10,463 (49.42)	
5.1–10	8,571 (20.24)	4,321 (20.41)	
>10	2,415 (5.70)	1,215 (5.74)	
Histological type			0.418
EEA	37,041 (87.48)	18,468 (87.24)	
SEA	3,185 (7.52)	1,621 (7.66)	
Other types	2,114 (4.99)	1,081 (5.11)	
Myometrial invasion			0.682
<50%	27,721 (65.47)	13,792 (65.14)	
≥50%	14,619 (34.53)	7,378 (34.86)	
Cervical stromal invasion			0.959
Negative	33,797 (79.82)	16,884 (79.75)	
Positive	8,543 (20.18)	4,286 (20.25)	
LNM			0.327
Negative	37,500 (88.57)	18,775 (88.69)	
Positive	4,840 (11.43)	2,395 (11.31)	
Grade			0.545
G1	17,748 (41.92)	8,838 (41.75)	
G2	12,691 (29.97)	6,262 (29.58)	
G3	11,901 (28.11)	6,070 (28.67)	
Stage			0.594
I	29,831 (70.46)	14,935 (70.55)	
II	3,458 (8.17)	1,677 (7.92)	
III	6,623 (15.64)	3,336 (15.76)	
IV	2,428 (5.73)	1,222 (5.77)	
Chemotherapy			0.082
No	34,276 (80.95)	17,026 (80.43)	
Yes	8,064 (19.05)	4,144 (19.57)	
Radiation			0.052
No	30,202 (71.33)	15,044 (71.06)	
Yes	12,138 (28.67)	6,126 (28.94)	
Overall survival			0.778
Alive	32,905 (77.72)	16,435 (77.63)	
Death	9,435 (22.28)	4,735 (22.37)	
Cancer-specific survival			0.972
Alive	35,695 (84.31)	17,791 (84.04)	
Death	6,645 (15.69)	3,379 (15.96)	

*EEA, endometrial endometrioid adenocarcinoma; SEA, serous endometrioid adenocarcinoma; LNM, lymph node metastasis. Marital status-others represent widowed and divorced. Chi-square or Fisher exact tests are used. There are no significant difference between the two cohorts for all of the p-value > 0.05. P-value <0.05 is taken to be significant*.

### Independent Prognostic Factors for OS and CSS

**Figure 2:**
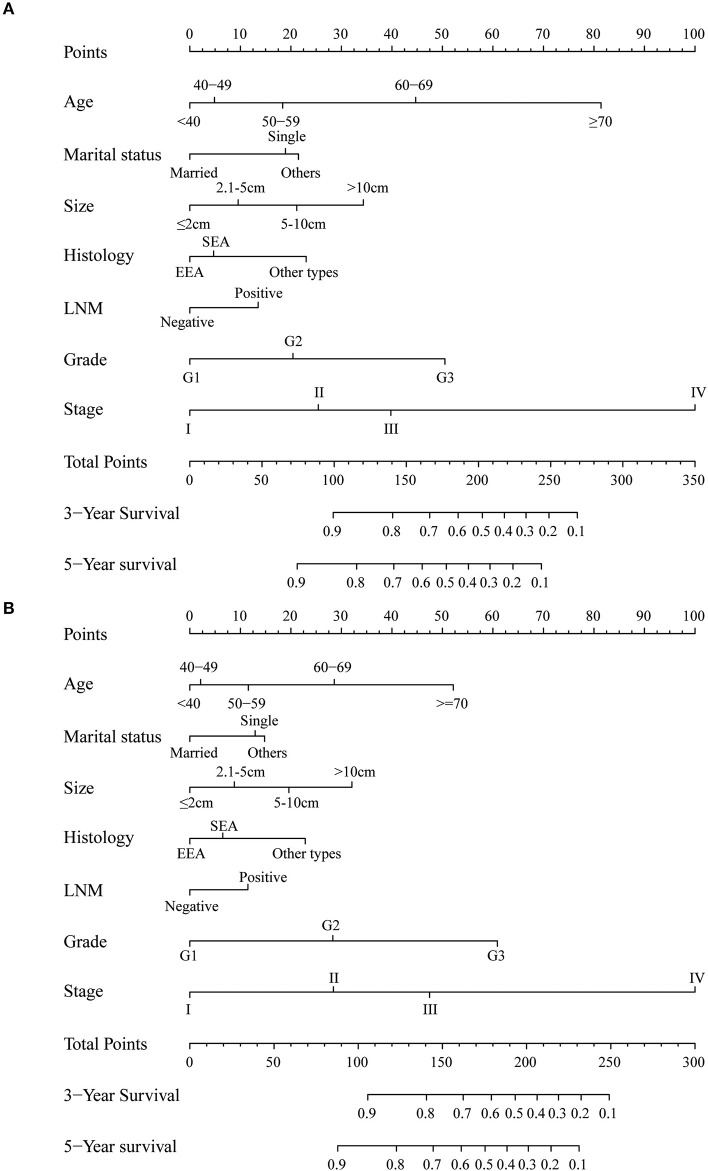
Nomogram to predict 3- and 5-year **(A)** overall survival and **(B)** cancer-specific survival for endometrial cancer patients. EEA, endometrial endometrioid adenocarcinoma; SEA, serous endometrioid adenocarcinoma; LNM, lymph node metastasis.

The univariate and multivariate analyses of potential predictors for the OS in the training group and the validation group are shown in Table 2. Age at diagnosis, marital status, tumor size, histological type, myometrial invasion, cervical stromal invasion, lymph node metastasis, tumor grade, clinical stage, chemotherapy, radiotherapy were significantly associated as risk factors for the OS according to univariate analysis for both the groups. For this reason, these risk factors were included in the multivariate analysis. Multivariate analysis identified that age at diagnosis (51–60, HR = 1.37, 95%CI: 1.15–1.63, *p* = 0.004; 61–70, HR = 2.17, 95%CI: 1.82–2.58, *p* < 0.001; >70, HR = 4.01, 95%CI: 3.37–4.77, *p* < 0.001), marital status (single, HR = 1.39, 95%CI: 1.31–1.47, *p* < 0.001; others, HR = 1.46, 95%CI: 1.39–1.52, *p* < 0.001), tumor size (2.1–5 cm, HR = 1.13, 95%CI: 1.06–1.20, *p* < 0.001; 5.1–10 cm, HR = 1.39, 95%CI: 1.30–1.49, *p* < 0.001; >10 cm, HR = 1.75, 95%CI: 1.60–1.92, *p* < 0.001;), histological type (SEA, HR = 1.13, 95%CI: 1.06–1.21, *p* < 0.001; other types, HR = 1.53, 95%CI: 1.43–1.64, *p* < 0.001), lymph node metastasis (HR = 1.36, 95%CI: 1.28–1.44, *p* < 0.001), tumor grade (G2, HR = 1.45, 95%CI: 1.36–1.54, *p* < 0.001; G3, HR = 2.62, 95%CI: 2.46–2.79, *p* < 0.001), clinical stage (stage II, HR = 1.24, 95%CI: 1.15–1.35, *p* < 0.001; stage III, HR = 1.73, 95%CI: 1.61–1.86, *p* < 0.001; stage IV, HR = 4.92, 95%CI: 4.54–5.34, *p* < 0.001) were independent risk factors to predict OS in the training group. The analysis of the validation group showed similar results (Table 2). These variables remained statistically significant (*p* < 0.01) according to the multivariate analysis for CSS (Table 3). For this reason, age at diagnosis, marital status, tumor size, histological type, lymph node metastasis, tumor grade, and clinical stage were identified as independent prognostic factors for both OS and CSS for the EC patients. Independent prognostic factors were used to develop the nomograms for 3- and 5-year OS and CSS (Figures 2A,B). Detailed points of each predictor in the nomograms were listed in Table 4. By including these scores to the total on the bottom scale, the 3- and 5-year OS and CSS can be predicted.

**Table 2:** Univariate and multivariate analysis of overall survival in training and validation cohorts.

	**Training cohort**				**Validation cohort**			
	**Univariate**		**Multivariate**		**Univariate**		**Multivariate**	
**Age at diagnosis(years)**	**HR (95%CI)**	***p*-value**	**HR (95%CI)**	***p*-value**	**HR (95%CI)**	***p*-value**	**HR (95%CI)**	***p*-value**
≤ 40	Reference		Reference		Reference		Reference	
41–50	1.13 (0.93, 1.37)	0.209	1.08 (0.89, 1.31)	0.410	1.00 (0.77, 1.29)	0.971	1.04 (0.80, 1.36)	0.754
51–60	1.47 (1.23, 1.74)	<0.001	1.37 (1.15, 1.63)	0.004	1.35 (1.06, 1.71)	0.014	1.36 (1.07, 1.73)	0.012
61–70	2.56 (2.16, 3.04)	<0.001	2.17 (1.82, 2.58)	<0.001	2.22 (1.76, 2.81)	<0.001	2.01 (1.58, 2.54)	<0.001
>70	5.83 (4.92, 6.92)	<0.001	4.01 (3.37, 4.77)	<0.001	4.95 (3.92, 6.24)	<0.001	3.78 (2.98, 4.79)	<0.001
**Marital status**								
Married	Reference		Reference		Reference		Reference	
Single	1.28 (1.21, 1.36)	<0.001	1.39 (1.31, 1.47)	<0.001	1.23 (1.14, 1.34)	<0.001	1.27 (1.17, 1.37)	<0.001
Others	2.07 (1.98, 2.17)	<0.001	1.46 (1.39, 1.52)	<0.001	2.06 (1.94, 2.20)	<0.001	1.39 (1.30, 1.48)	<0.001
**Tumor size (cm)**								
≤ 2	Reference		Reference		Reference		Reference	
2.1–5	1.66 (1.56, 1.76)	<0.001	1.13 (1.06, 1.20)	<0.001	1.67 (1.53, 1.82)	<0.001	1.14 (1.05, 1.25)	0.003
5.1–10	3.10 (2.91, 3.31)	<0.001	1.39 (1.30, 1.49)	<0.001	3.23 (2.95, 3.54)	<0.001	1.54 (1.40, 1.70)	<0.001
>10	3.24 (2.98, 3.53)	<0.001	1.75 (1.60, 1.92)	<0.001	3.28 (2.91, 3.71)	<0.001	1.90 (1.67, 2.16)	<0.001
**Histological type**								
EEA	Reference		Reference		Reference		Reference	
SEA	3.22 (3.04, 3.41)	<0.001	1.13 (1.06, 1.21)	0.001	3.20 (2.95, 3.47)	<0.001	1.12 (1.03, 1.22)	0.010
Other types	3.14 (2.94, 3.35)	<0.001	1.53 (1.43, 1.64)	<0.001	2.98 (2.71, 3.27)	<0.001	1.43 (1.29, 1.57)	<0.001
**Myometrial invasion**								
<50%	Reference				Reference			
≥50%	3.18 (2.96, 3.43)	<0.001			2.83 (2.56, 3.12)	<0.001		
**Cervical invasion**								
Negative	Reference				Reference			
Positive	3.74 (3.58, 3.91)	<0.001			3.76 (3.53, 4.00)	<0.001		
**LNM**								
Negative	Reference		Reference		Reference		Reference	
Positive	4.08 (3.90, 4.27)	<0.001	1.36 (1.28, 1.44)	<0.001	4.12 (3.86, 4.39)	<0.001	1.27 (1.17, 1.38)	<0.001
**Tumor grade**								
G1	Reference		Reference		Reference		Reference	
G2	1.98 (1.86, 2.10)	<0.001	1.45 (1.36, 1.54)	<0.001	1.85 (1.69, 2.01)	<0.001	1.36 (1.25, 1.49)	<0.001
G3	5.88 (5.57, 6.20)	<0.001	2.62 (2.46, 2.79)	<0.001	5.85 (5.42, 6.31)	<0.001	2.45 (2.24, 2.67)	<0.001
**Stage**								
I	Reference		Reference		Reference		Reference	
II	2.11 (1.97, 2.27)	<0.001	1.24 (1.15, 1.35)	<0.001	2.10 (1.90, 2.34)	<0.001	1.24 (1.11, 1.40)	0.003
III	3.67 (3.49, 3.86)	<0.001	1.73 (1.61, 1.86)	<0.001	3.88 (3.62, 4.15)	<0.001	1.71 (1.55, 1.90)	<0.001
IV	13.17 (12.45, 13.93)	<0.001	4.92 (4.54, 5.34)	<0.001	14.07 (13.00, 15.23)	<0.001	5.04 (4.52, 5.61)	<0.001
**Chemotherapy**								
No	Reference				Reference			
Yes	2.82 (2.70, 2.94)	<0.001			2.71 (2.55, 2.88)	<0.001		
**Radiation**								
No	Reference				Reference			
Yes	1.34 (1.28, 1.39)	<0.001			1.32 (1.24, 1.40)	<0.001		

*HR, Hazard Ratio; CI, Confidence Interval. EEA, endometrial endometrioid adenocarcinoma; SEA, serous endometrioid adenocarcinoma; LNM, lymph node metastasis. Univariate and multivariate Cox regression analyses are used. P-value <0.05 is taken to be significant*.

**Table 3:** Univariate and multivariate analysis of cancer-specific survival in training and validation cohorts.

	**Training cohort**				**Validation cohort**			
	**Univariate**		**Multivariate**		**Univariate**		**Multivariate**	
**Age (years)**	**HR (95%CI)**	***p*-value**	**HR (95%CI)**	***p*-value**	**HR (95%CI)**	***p*-value**	**HR (95%CI)**	***p*-value**
≤ 40	Reference		Reference		Reference		Reference	
41–50	1.12 (0.91, 1.39)	0.291	1.09 (0.90, 1.32)	0.392	1.03 (0.76, 1.38)	0.867	1.04 (0.77, 1.40)	0.793
51–60	1.43 (1.17, 1.74)	0.004	1.36 (1.14, 1.62)	0.006	1.33 (1.01, 1.74)	0.041	1.26 (0.96, 1.66)	0.096
61–70	2.31 (1.90, 2.80)	<0.001	2.17 (1.82, 2.57)	<0.001	2.12 (1.62, 2.76)	<0.001	1.71 (1.30, 2.24)	0.001
>70	4.57 (3.77, 5.54)	<0.001	4.15 (3.49, 4.93)	<0.001	4.05 (3.11, 5.28)		2.68 (2.04, 3.52)	<0.001
**Marital status**								
Married	Reference		Reference		Reference		Reference	
Single	1.27 (1.19, 1.36)	<0.001	1.41 (1.33, 1.49)	<0.001	1.16 (1.05, 1.28)	0.002	1.12 (1.02, 1.24)	0.021
Others	1.86 (1.77, 1.97)	<0.001	1.48 (1.41, 1.54)	<0.001	1.91 (1.77, 2.05)	<0.001	1.30 (1.21, 1.41)	<0.001
**Tumor size (cm)**								
≤ 2	Reference		Reference		Reference		Reference	
2.1–5	1.82 (1.69, 1.97)	<0.001	1.11 (1.04, 1.18)	0.001	1.75 (1.57, 1.95)	<0.001	1.10 (0.98, 1.22)	0.104
5.1–10	3.99 (3.68, 4.32)	<0.001	1.34 (1.26, 1.44)	<0.001	3.98 (3.57, 4.45)	<0.001	1.51 (1.35, 1.70)	<0.001
>10	4.52 (4.09, 5.00)	<0.001	1.75 (1.60, 1.91)	<0.001	4.41 (3.83, 5.08)	<0.001	1.92 (1.65, 2.22)	<0.001
**Histological type**								
EEA	Reference		Reference		Reference		Reference	
SEA	4.05 (3.80, 4.31)	<0.001	1.10 (1.04, 1.17)	0.002	4.01 (3.67, 4.38)	0.001	1.17 (1.06, 1.29)	0.001
Other types	4.05 (3.76, 4.36)	<0.001	1.52 (1.42, 1.63)	<0.001	3.73 (3.36, 4.15)	0.001	1.50 (1.35, 1.67)	<0.001
**Myometrial invasion**								
<50%	Reference				Reference			
≥50%	4.11 (3.72, 4.54)	<0.001			4.03 (3.51, 4.63	<0.001		
**Cervical invasion**								
Negative	Reference				Reference			
Positive	5.26 (4.97, 5.57)	<0.001			5.35 (4.93, 5.80)	<0.001		
**LNM**								
Negative	Reference		Reference		Reference		Reference	
Positive	5.42 (5.15, 5.71)	<0.001	1.31 (1.23, 1.39)	<0.001	5.42 (5.04, 5.82)	<0.001	1.29 (1.18, 1.41)	<0.001
**Grade**								
G1	Reference		Reference		Reference		Reference	
G2	2.65 (2.43, 2.88)	<0.001	1.39 (1.31, 1.48)	<0.001	2.34 (2.08, 2.63)	0.001	1.64 (1.46, 1.85)	<0.001
G3	10.16 (9.43, 10.95)	<0.001	2.39 (2.25, 2.55)	<0.001	9.76 (8.81, 10.82)	0.001	3.50 (3.12, 3.93)	<0.001
**Stage**								
I	Reference		Reference		Reference		Reference	
II	2.60 (2.38, 2.85)	<0.001	1.19 (1.10, 1.29)	<0.001	2.67 (2.35, 3.03)	<0.001	1.42 (1.23, 1.64)	<0.001
III	5.57 (5.24, 5.91)	<0.001	1.59 (1.48, 1.71)	<0.001	5.88 (5.41, 6.39)	<0.001	2.20 (1.95, 2.49)	<0.001
IV	21.67 (20.33, 23.11)	<0.001	4.68 (4.34, 5.06)	<0.001	22.45 (20.50, 24.58)	<0.001	6.55 (5.78, 7.43)	<0.001
**Chemotherapy**								
No	Reference				Reference			
Yes	3.94 (3.76, 4.14)	<0.001			3.75 (3.50, 4.01)	<0.001		
**Radiation**								
No	Reference				Reference			
Yes	1.47 (1.39, 1.54)	<0.001			1.46 (1.36, 1.57)	<0.001		

*HR, Hazard Ratio; CI, Confidence Interval. EEA, endometrial endometrioid adenocarcinoma; SEA, serous endometrioid adenocarcinoma; LNM, lymph node metastasis. Univariate and multivariate Cox regression analyses are used. P-value <0.05 is taken to be significant*.

**Table 4:** Detailed score of each prognostic factor in overall and cancer-specific survival nomograms.

**Characteristics**	**OS nomogram**	**CSS nomogram**
**Age at diagnosis (year)**		
≤ 40	0	0
41–50	5	2.5
51–60	18	12
61–70	45	28
>70	82	52.5
**Marital status**		
Married	0	0
Single	18	13
Others	21	15
**Tumor size (cm)**		
≤ 2	0	0
2.1–5	10	8
5.1–10	20	20
>10	33	30
**Histological type**		
EEA	0	0
SEA	5	7.5
Other types	23	23
**LNM**
Negative	0	0
Positive	15	12.5
**Tumor grade**		
G1	0	0
G2	20	28
G3	50	60
**Stage**		
I	0	0
II	25	28
III	40	47
IV	100	100

*OS, overall survival; CSS, cancer-specific survival. EEA, endometrial endometrioid adenocarcinoma; SEA, serous endometrioid adenocarcinoma; LNM, lymph node metastasis*.

### Calibration and Discrimination of the Nomograms

**Figure 3:**
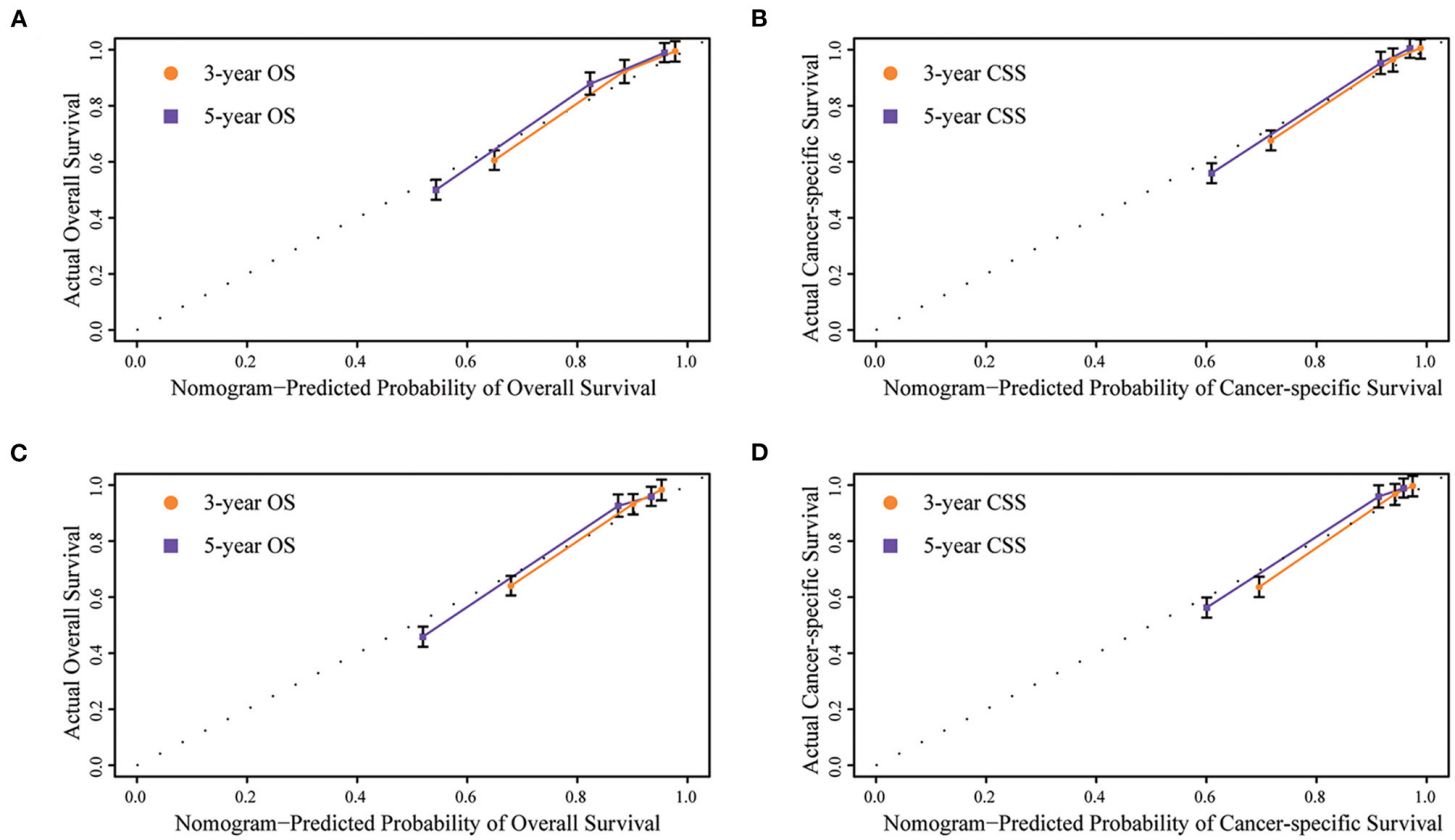
Internal calibration plots of **(A)** 3- and 5-year overall survival in training group, **(B)** 3- and 5-year cancer-specific survival in training group, **(C)** 3- and 5-year overall survival in validation group, **(D)** 3- and 5-year cancer-specific survival in validation group. The dashed line represents an excellent match between nomogram predicted survival (X-axis) and actual survival outcome (Y-axis). A plot along the dashed line indicates a perfect calibration model.

**Figure 4:**
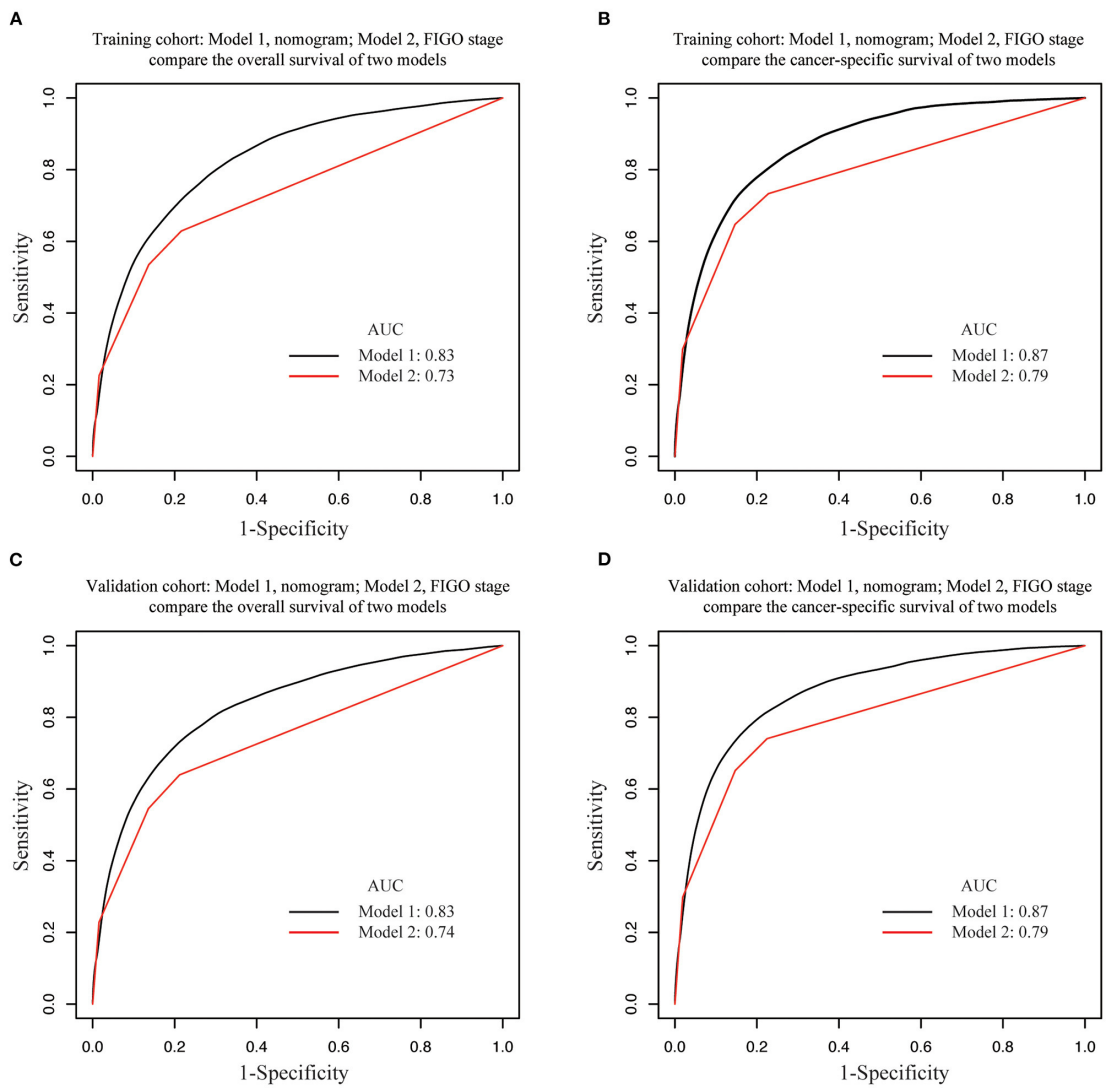
Area under the receiver operating characteristic curves (AUCs) of nomograms and FIGO staging system for the prediction of overall survival (OS) and cancer-specific survival (CSS) in EC in training and validation cohorts. AUCs comparison in **(A)** OS in training cohort, **(B)** CSS in training cohort, **(C)** OS in validation cohort, **(D)** CSS in validation cohort. Model 1, nomogram; Model 2, FIGO staging system.

Internal and external validations of the prognostic nomograms were performed. Internal validation in the training cohort showed that the C-index values for nomogram predictions of OS and CSS were 0.816 (95%CI: 0.811–0.820) and 0.850 (95%CI: 0.846–0.855), respectively. Similarly, the corresponding C-index values in the external validation cohort were 0.812 (95%CI: 0.806–0.819) and 0.847 (95%CI: 0.841–0.854). These results confirmed that our prognostic nomograms were reasonably accurate. The calibration curves estimating the 3- and the 5-year OS and CSS rates showed accordance between the nomogram-predicted and observed values in both the training and the validation groups (Figure 3). Furthermore, predictive accuracy and differentiation of patients were compared between the nomogram and the clinical stage system. In the training group, the AUC of the established nomogram (AUC = 0.83, 95%CI: 0.71–0.87) to predict OS was significantly higher than that of the clinical stage (AUC = 0.73, 95%CI: 0.67–0.80, *p* < 0.01; Figure 4A). While for the CSS, the AUC of the nomogram (AUC = 0.87, 95%CI: 0.85–0.90) was also greater than that of the clinical stage (AUC = 0.79, 95%CI: 0.71–0.84, *p* < 0.01; Figure 4B). The analysis of the validation group showed similar results (Figures 4C,D). The nomograms for OS and CSS showed superior discrimination as compared to the clinical stage both in the training and the validation cohorts.

### Performance of the Nomogram in Stratifying the Risk for EC Patients

**Figure 5:**
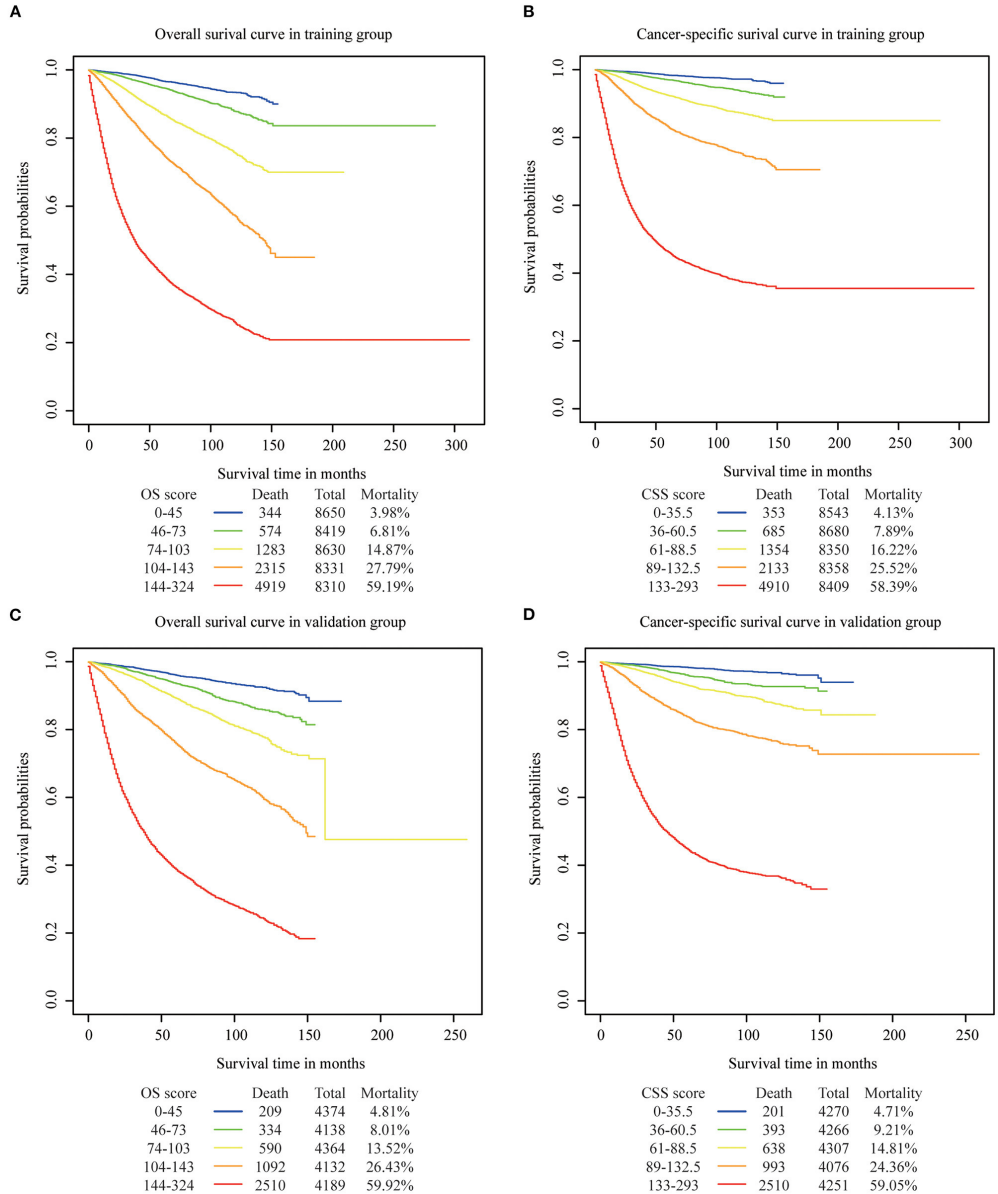
Overall and cancer specific survival in different risk stratifications. Patients were divided into five groups according to their total OS or CSS score. **(A)** OS in training cohort, **(B)** CSS in training cohort, **(C)** OS in validation cohort, **(D)** CSS in validation.

**Figure 6:**
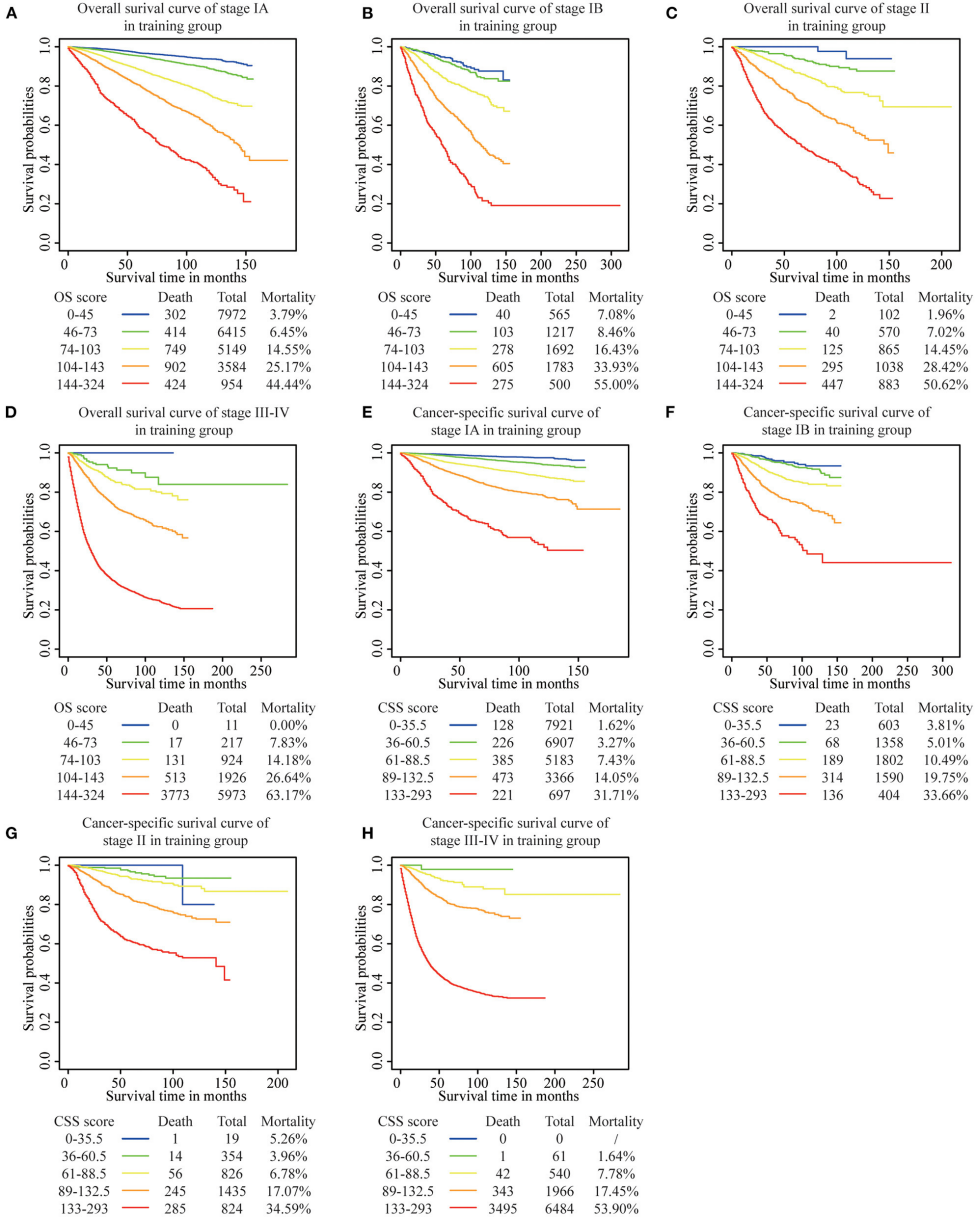
Risk group stratification within each FIGO staging system in the training group. **(A)** OS in stage IA, **(B)** OS in stage IB, **(C)** OS in stage II, **(D)** OS in stage III-IV, **(E)** CSS in stage IA, **(F)** CSS in stage IB, **(G)** CSS in stage II, **(H)** CSS in stage III-IV.

We determined the cutoff values by dividing the patients in the training cohort evenly into five subgroups after sorting by the total OS score (score: 0–45, 46–73, 74–103, 104–143, 144–342) and the total CSS score (score: 0–35.5, 36–60.5, 61–88.5, 89–132.5, 133–293), and each group represented a distinct prognosis (Figure 5). As shown in Figure 5A, the mortality of patients in the low-score group was 3.98%, and reached the 59.19% value in the high-score group. After applying the cutoff values to the groups of patients in the different clinical staging cohorts, stratification into different risk subgroups in both the training and the validation groups ensured a significant distinction between Kaplan-Meier curves for OS and the CSS outcomes within each clinical stage category (Figure 6 and Supplementary Figure 1). For patients in stage IA, the overall survival was very different in the different subgroups. As an example, in patients in stage IA whose OS score was >144, the mortality was as high as 44.44% (Figure 6A), and patients in stage III-IV whose OS score was <73, the mortality was lower than 7.83% (Figure 6D). Using this methodology, we could not only select high-risk patients at low stages, but also distinguish low-risk patients at high stages.

## Discussion

The clinical staging system is the most common method to predict the prognosis in endometrial cancer. The 2009 FIGO staging system included many of the known risk factors for OS and CSS, such as myometrial invasion, cervical stromal invasion, and lymph node metastasis. However, other important risk factors including age at diagnosis, tumor grade, and histologic type that may affect the survival, are not currently included in the FIGO system. Furthermore, in order to predict the individual survival probability, a single prognostic index may impose limitations on the individual prognosis. Accurate individual prognosis helps to avoid oncological under-treatment or over-treatment in EC patients.

Nomogram is a common graphic prediction tool that can provide a more precise and acceptable accuracy and robustness to predict an individual patient's survival probability. In this study, we developed prognostic nomograms to predict the OS and CSS using univariate and multivariate Cox analyses by a large population-based database. Several clinicopathological characteristics have proven to be independent from other risk factors such as age at diagnosis, marital status, tumor size, histological type, lymph node metastasis, tumor grade, and clinical stage. Our nomograms identified these seven independent risk factors to determine the survival of EC patients and provided with an internal validation for the nomograms predicting the 3- and the 5-year OS and CSS in EC patients. Abu-Rustum constructed the first nomogram based on 5 variables to predict the OS and the study was based on 1,735 patients who received the treatment at the MSKCC in New York City (12). The internal validation showed a C-index of 0.75, which represented an acceptable value for the patient differentiation between the subjects who died as compared to those who survived. However, the authors did not include the marital status and the tumor size as additional risk factors. A previous study demonstrated that married women affected by EC showed a favorable diagnosis and prognosis (16). We observed a similar trend also in our study where single women had a higher risk of mortality as compared to married women.

A previous study demonstrated that large tumor size was an important risk factor for a multitude of tumors including bladder (17), lung (18), and prostate cancers (19). Our study suggested that patients with larger tumors had a diminished survival as compared to those with smaller size tumors. Koskas et al. and Polterauer et al. validated the Abu-Rustum's nomogram using an external cohorts, and proved that the nomogram could accurately predict the 3-year overall survival (20, 21). They also found that the nomogram was sufficient even for EC patients receiving adjuvant radiotherapy or not. Radiotherapy was found to be an independent risk factor for breast cancer and prostate cancer (22, 23). The reason why adjuvant therapy was not included in Abu-Rustum's and our nomogram might be that the patients who underwent adjuvant radiotherapy and adjuvant chemotherapy were older and had an higher histological grade. Additionally, adjuvant therapy was only recommended for a subset of patients who had potentially high risk of disease recurrence. The adjuvant therapies, when included into the nomogram, have a contradictory impact on the treated and untreated populations, determining the assignment of a wrong score to the untreated patients. Compared with the MSKCC nomogram, our nomogram has been developed from a relatively larger cohort and predicted not only the overall survival but also the cancer-specific survival. The determined C-index for our OS nomogram is 0.816, which is greater than any of the MSKCC internal validation or external groups.

The validation of the nomogram is essential to avoid overfitting of the model and determine the scope of its application. In this study, calibration plots revealed an ideal accordance between the prediction and the actual observation in both the training and the validation groups, which illustrated the repeatability and reliability of the OS and the CSS nomograms. Patient differentiation was determined through a significantly higher AUC of the nomogram as compared to the FIGO staging system in both the cohorts. Historically, the FIGO staging system helped to standardize the therapeutic management and predict the OS. Our prognostic nomograms attempted to combine the FIGO staging system with other determinant clinical factors in order to predict the individual's survival outcome in all EC stages. Therefore, our nomogram represented a more accurate individual performance predicting prognosis as compared to the 2009 FIGO staging systems for EC. Our study revealed that the AUCs of the nomograms for OS and CSS were both greater than the AUCs as determined by the FIGO staging system, suggesting that the nomograms were more accurate in predicting the OS and CSS in EC patients. We also found that the MSKCC nomogram was more accurate than the 2009 FIGO staging system in predicting the OS (24).

For a more detailed validation of the nomograms, we calculated the total scores for each patient and divided the patients into 5 subgroups (OS score: 0–45, 46–73, 74–103, 104–143, 144–342; CSS score: 0–35.5, 36–60.5, 61–88.5, 89–132.5, 133–293) according to the total scores. According to our data, the survival rate of patients in the low score subgroup was always higher than patients belonging to the high score group, either for the OS or the CSS. The scoring system could also precisely differentiate patients in the same clinical stage but with a worse prognosis, which could be determinant in the context of an individualized treatment and of the precise therapy.

This study constructed a nomogram predicting both OS and CSS based on a relative large population with a long term follow-up, and the nomogram outperformed in different layers of clinical characteristics. However, some limitations might affect our study. First, we developed and validated the nomograms using retrospective data. Although we collected the largest cohort using the SEER database which represented ~30% of the US population, the nomograms needed to be validated in a randomized controlled trial for a better reliability. The second limitation was that several recognized prognostic parameters, such as lymph-vascular space invasion (LVSI), serum CA-125 level, and chronic disease history, were unavailable in the SEER database. The external validation of the nomogram in this study also needed to be explored using prospective datasets.

## Conclusion

Above all, we developed and validated nomograms which predicted the 3- and the 5-year OS and CSS in EC patients based on a large, population-based cohort. The nomogram demonstrated a higher predictive accuracy than the FIGO staging system. Through this model, gynecologic oncologists could estimate the survival of individual patients more precisely and identify subgroups of patients needing a specific treatment strategy.

## Data Availability Statement

The original contributions presented in the study are included in the article/Supplementary Materials, further inquiries can be directed to the corresponding author/s.

## Ethics Statement

Ethical review and approval was not required for the study on human participants in accordance with the local legislation and institutional requirements. Written informed consent for participation was not required for this study in accordance with the national legislation and the institutional requirements.

## Author Contributions

JW and XinL: conceptualization. YF and YD: methodology. YF: software. YD and YC: formal analysis. JZ: investigation. XiaL: resources. ZW: data curation. XinL: writing – original draft preparation. YF and JW: writing – review and editing. JW and YC: funding acquisition. All authors contributed to the article and approved the submitted version.

## Conflict of Interest

The authors declare that the research was conducted in the absence of any commercial or financial relationships that could be construed as a potential conflict of interest.

## Supplementary Material

Supplementary Figure 1 Risk group stratification within each FIGO staging system in the validation group. **(A)** OS in stage IA, **(B)** OS in stage IB, **(C)** OS in stage II, **(D)** OS in stage III-IV, **(E)** CSS in stage IA, **(F)** CSS in stage IB, **(G)** CSS in stage II, **(H)** CSS in stage III-IV.

## Abbreviations

OS: overall survival
CSS: cancer-specific survival
EC: endometrial cancer
SEER, Surveillance, Epidemiology: and End Results database
C-index: concordance index
ROC, receiver operating characteristics, FIGO: International Federation of Gynecology and Obstetrics
MSKCC: Memorial Sloan Kettering Cancer Center
EEA: endometrial endometrioid adenocarcinoma
SEA. serous endometrioid adenocarcinoma
AUC: area under the ROC curve
HR, hazard ratios, CI: confidence interval.
