# Supplementary figures and images for: Development and Validation of Nomograms Predicting the Overall and the Cancer-Specific Survival in Endometrial Cancer Patients
Source: Front Med (Lausanne). 2020 Dec 23;7:614629. doi: 10.3389/fmed.2020.614629 (PMC7785774; doi:10.3389/fmed.2020.614629)

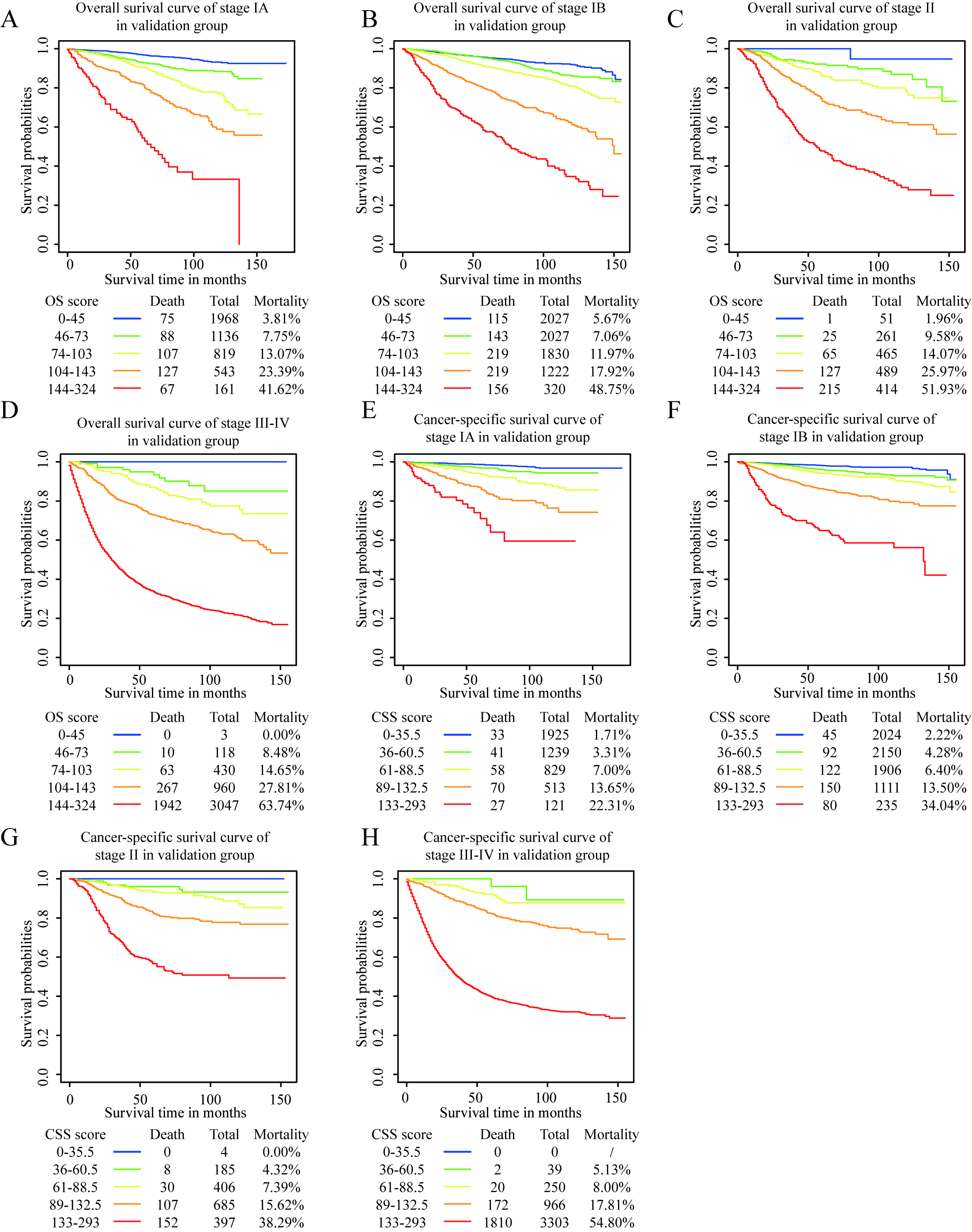

Supplement: Supplementary Figure 1 — Risk group stratification within each FIGO staging system in the validation group. (A) OS in stage IA, (B) OS in stage IB, (C) OS in stage II, (D) OS in stage III-IV, (E) CSS in stage IA, (F) CSS in stage IB, (G) CSS in stage II, (H) CSS in stage III-IV. [file Image_1.TIF]
